# Hand hygiene campaign to all the residential care home for elderly (RCHE) in Hong Kong

**DOI:** 10.1186/2047-2994-4-S1-P160

**Published:** 2015-06-16

**Authors:** J Leung, A Wong, T Wong

**Affiliations:** 1 Infection Control Branch, Centre for Health Protection, Department of Health, Hong Kong SAR, China

## Introduction

Multidrug Resistant Organisms (MDROs) is a global public health challenge. *Vancomycin Resistant Enterococcus*(VRE) is a type of MDRO. There is a surge of incidence of infection and colonization with VRE in Hong Kong in 2013. Outbreaks caused by VRE occurred in hospitals; and previous experience showed that elderly population were at high risk. Infection Control Branch (ICB) anticipated that there might be increased involvement of RCHE residents following the pan-screening exercise in a public hospital. In an effort to prevent the spread of VRE within the RCHEs, a territory-wide hand hygiene campaign to all RCHEs was initiated in late 2013.

## Objectives

To review the local situation of prevention and control of infectious diseases in RCHEs in Hong Kong and to develop a local action plan to enhance hand hygiene compliance among RCHEs in Hong Kong.

## Methods

A locally adapted hand hygiene program for RCHE was developed based on the WHO guidelines on hand hygiene in health care. The program consists of 5 important components: System Change, Training / Education, Evaluation and feedback, Reminders in the workplace, Institutional safety climate.

## Results

A total of 2400 RCHE staff received the training in the campaign. From 17 September 2013 to 27 September 2013, 96% of the RCHEs in Kowloon have been visited. By the end of October 2013, more than 90% of the RCHEs territory-wide have received the targeted training. Simplified education kit including posters and gimmicks were delivered to all RCHEs in October.

## Conclusion

A multimodal WHO framework comprehensive hand hygiene promotion programme was successfully implemented. The program contributes not only hand hygiene improvement in RCHE but also psychological acceptances for caring VRE residents in RCHE.

## Disclosure of interest

None declared.

